# Quantitative X-ray Tomography of the Mouse Cochlea

**DOI:** 10.1371/journal.pone.0033568

**Published:** 2012-04-02

**Authors:** Christoph Rau, Margaret Hwang, Wah-Keat Lee, Claus-Peter Richter

**Affiliations:** 1 Diamond Light Source Ltd., Diamond House, Harwell Science and Innovation Campus, Didcot, United Kingdom; 2 Department of Otolaryngology, Northwestern University Feinberg School of Medicine, Chicago, Illinois, United States of America; 3 Advanced Photon Source, Argonne National Laboratory, Argonne, Illinois, United States of America; 4 Department of Biomedical Engineering, Northwestern University, Evanston, Illinois, United States of America; 5 The Hugh Knowles Center, Department of Communication Sciences and Disorders, Northwestern University, Evanston, Illinois, United States of America; University of Rochester, United States of America

## Abstract

Imaging with hard X-rays allows visualizing cochlear structures while maintaining intrinsic qualities of the tissue, including structure and size. With coherent X-rays, soft tissues, including membranes, can be imaged as well as cells making use of the so-called in-line phase contrast. In the present experiments, partially coherent synchrotron radiation has been used for micro-tomography. Three-dimensional reconstructions of the mouse cochlea have been created using the EM3D software and the volume has been segmented in the Amira Software Suite. The structures that have been reconstructed include scala tympani, scala media, scala vestibuli, Reissner's membrane, basilar membrane, tectorial membrane, organ of Corti, spiral limbus, spiral ganglion and cochlear nerve. Cross-sectional areas of the scalae were measured. The results provide a realistic and quantitative reconstruction of the cochlea.

## Introduction

Today, mice provide the most common model to study hereditary abnormalities of the human cochlea because the homologies between mice and human genomes are well established and the hereditary abnormalities are similar [1], [2]. Genetically induced abnormalities might manifest themselves in changes of cochlear dimensions and thus disturb normal cochlear mechanics and cochlear function. Therefore, it is of special interest to study mice with known gene defects and compromised cochlear function regarding anatomical and histological changes. Current techniques for using series of images to obtain a three-dimensional structure of the cochlear anatomy include classical histology (sectioning and staining), magnetic resonance imaging (MRI), high-resolution computed tomography (CT) using conventional X-ray sources and synchrotron radiation [3], orthogonal-plane optical fluorescence [4]–[8], and optical coherence tomography (OCT) using lasers [9]–[15]. A three-dimensional model of the mouse cochlea has been created using magnetic resonance microscopy [8].

Sectioning of a cochlea involves several steps, such as fixation, dehydration, embedding, and slicing of the sample. These steps may significantly change the dimension of the soft tissues [16]–[18], including Reissner's membrane.

Knowing the true location of Reissner's membrane also has clinical utility. For example, the bulging of Reissner's membrane, which has been shown in Ménière's disease [19], may disappear through shrinkage. Moreover, reconstruction of altered tissue may provide different volumes of scala vestibuli, scala tympani, and scala media [17].

MRI is a powerful method for investigating the cochlea but is intrinsically slower at higher resolutions and even then cannot resolve all structures of interest. Often, MRI is supplemented by high-resolution CT [6]. Today, high-resolution CT with laboratory sources typically achieves a resolution of approximately 10 µm and provides a more efficient way to view the cochlea. However, for cellular or sub-cellular structures, including Reissner's membrane, the resolution of this technique, as implemented, is not sufficient.

Recently, it has been suggested that imaging with coherent hard X-rays from a synchrotron constitutes an ideal method for rapid characterization of cochlear morphology [20]–[22]. The method uses what is described as in-line phase contrast. The advantage of using hard X-rays and phase contrast to image the structures is the non-invasiveness, the spatial resolution, and the short time to acquire the images [22]–[25]. Generally, the method does not require dehydration, sectioning, or staining of the tissue, as is described in more detail in the following paragraphs.

## Materials and Methods

### The radiation source

For the present study, the X-ray images were captured at the 32-ID beamline of the Advanced Photon Source (APS) at Argonne National Laboratory. The APS is a third generation synchrotron radiation source that generates highly coherent X-ray radiation. The X-ray beam is generated with an undulator, inserted in a straight section of the storage ring. The full width half maximum (FWHM) electron source size during the reduced-horizontal-beta operational mode is 275 µm by 40 µm (horizontal × vertical) with an angular divergence of 60 µrad by 9 µrad (horizontal × vertical), FWHM. The monochromatic flux is about 10^13^ Photons/second @ 8–25 keV with a Si(111) double-crystal monochromator. The experimental setup was ∼70 m from the source and the beam size was approximately 1×2 mm. Measurements here were made using 25 keV X-rays with a sample-detector distance of about 0.8 m for phase-contrast. The typical detector system to capture the projections is described elsewhere [20] and provides a resolution of about 3 µm in the present study.

### Dissection and mounting of cochlear tissue

Animal care and use was carried out in accordance with the NIH Guide for the Care and Use of Laboratory Animals and was approved by the Northwestern University Animal Care and Use Committee.

For imaging, temporal bone preparations were made as follows. After an intraperitoneal injection of a lethal dose of sodium pentobarbital, mice (C57BL6), 12 weeks of age, were sacrificed. Following decapitation, the head was divided in the medial plane and the bullae were removed. The cochleae were excised, fixed with 4% paraformaldehyde in 0.1 M phosphate buffered solution (PBS), and then placed for one day into 10% ethylene glycol tetraacetic acid (EGTA) in PBS (0.1 M) for partial decalcification. In the present experiments the specimens were partially decalcified to reduce the X-ray absorption by the cochlear wall, and to ease the detection of the cochlear soft tissue structures. Fixation was used to stabilize the specimen during the decalcification (see also Discussion).

### Image acquisition and processing

A series of phase contrast X-ray projections was taken over a range of 180 degrees at increments of 1 degree. The exposure time for a single image was 0.6 s. At the end of each image series a flat field image (no object in the beam path) and a dark field image (the radiation beam was blocked) were captured. The raw images were corrected with the dark- and the flat-field and were scaled according to:
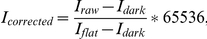
in which *I_corrected_* denotes the processed image, *I_raw_* the original image, *I_flat_* the flat field, *I_dark_* the dark field, and 65536 the maximum value to rescale the 16 bit-image.

For the reconstruction of the mouse cochlea with EM3D, the data (images of 1280 pixels horizontal ×1024 pixels vertical) were binned 2×2. The rotation axis was off the image center and was taken into account for the data manipulation. For each picture row the series of projections under different angles were used to reconstruct a slice of the cochlea. The software uses the filtered backprojection algorithm. This set of slices was used to render a three dimensional volume of the cochlea. The different cochlear structures were be segmented using the Amira Software, which utilizes a semi-automated segmentation algorithm. In the first step, the segmentation was achieved automatically based solely on the gray values of the image data set. This step separated the object from the background. This is done by segmenting the volume into exterior and interior regions on the basis of the voxel values. Each voxel having a value lower than the threshold is assigned to *Exterior* and each voxel whose value is greater than or equal to the threshold is assigned to *Interior*. This may, however, cause artifacts that are not part of the object, but which have voxel values above the threshold to be assigned to the interior. This can be suppressed by setting the *remove couch* option, which assures that only the largest coherent area will be labeled as the interior and all other voxels are assigned to the exterior. Furthermore, by inspecting the image, the segmentation was corrected manually. Not every slice was segmented. The software provided the option to project along the stack automatically. The segmentation results by the program were checked slice by slice and were corrected if necessary.

To determine the length of the basilar membrane, the heads of the pillar cells were identified and marked in the reconstructed slices. A line was drawn along the selected points and the program estimated its length in pixels. The pixel value was converted to µm with a conversion factor of 1.22 µm per pixels, which was determined with a calibration grid. Cross-sectional areas were determined by the software in a plane perpendicular to the line drawn at the pillar heads.

## Results

### Reconstructions

**Figure 1:**
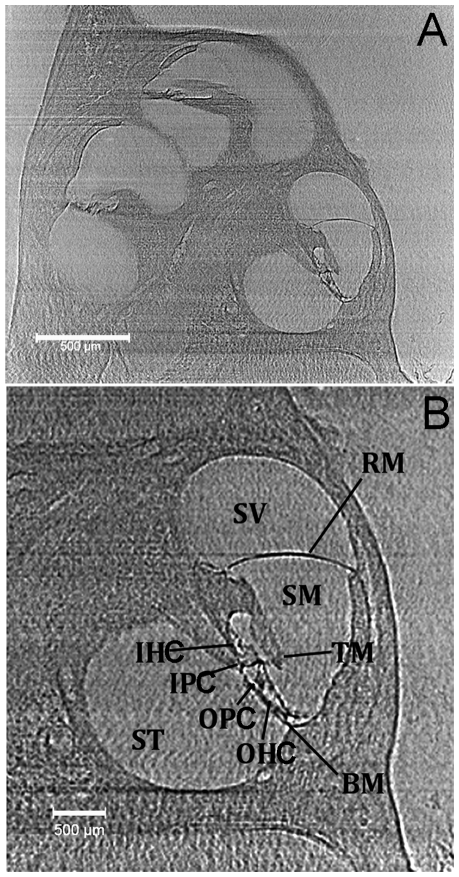
2D slice from cochlear reconstruction. 1A: 2D slice of the reconstruction. The image shows three turns of the mouse cochlea, including scala vestibuli, scala media, scala tympani, Reissner's membrane, tectorial membrane, organ of Corti, basilar membrane and stria vascularis. 1B: Magnified view of a cochlear turn. Clearly demonstrated and labeled are the scala vestibuli (SV), scala media (SM), scala tympani (ST), Reissner's membrane (RM), tectorial membrane (TM), basilar membrane (BM), outer hairs cells (OHC), inner hair cells (IHC), outer pillar cells (OPC), and inner pillar cells (IPC).

The stack of reconstructed slices was cut in an xy- and yz-plane, which are perpendicular to each other. Figure 1 shows a cut along the xz-plane. From base to apex, three cut edges can be identified. The image shows the cochlear wall, modiolus, basilar membrane, tectorial membrane, Reissner's membrane and stria vascularis. The edges of the structures, including soft tissues, are enhanced. Some horizontal lines and the granular structure in the background are image artifacts, which make the segmentation more difficult. Pillar heads were connected in Amira. The resulting line along the cochlea gave the length measurement of the cochlea (see also below). Cross sections of the reconstruction were made perpendicular to this line. Note for the following paragraph that interfaces between membranes, scalae, and bones can be clearly identified despite the noisy background.

### Segmenting and rendering

The xy-plane or the xz-plane is preferred for sectioning the data, since the edges of the structures can be clearly identified. This is not the case on the reconstructed slices (yz-plane), where the intersection angles between the membranes and the slices are rather shallow. In this case, the interfaces are more difficult to identify.

Figures 1A demonstrates that a small section at the cochlear base could not be imaged because the cochlea extended beyond the field of view. Area measurements at this location were not considered.

**Figure 2:**
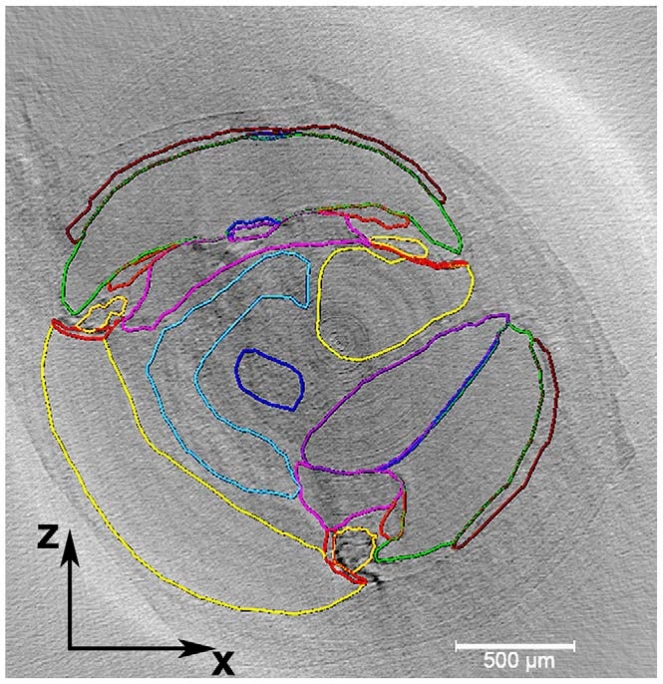
Segmentation of the cochlea. An example of the segmentation seen in the xz-plane. The colored segments (cochlear compartments) were filled in the gray slices using semi-automated algorithm in the Amira software. The purple denotes scala tympani and the green denotes scala media. The yellow denotes scala vestibuli. The periwinkle is spiral ganglion and the dark blue is cochlear nerve. The red is basilar membrane, the light blue is Reissner's membrane, the orange is tectorial membrane, and the goldenrod is organ of Corti. The pink is spiral limbus and brown stria vascularis.

**Figure 3:**
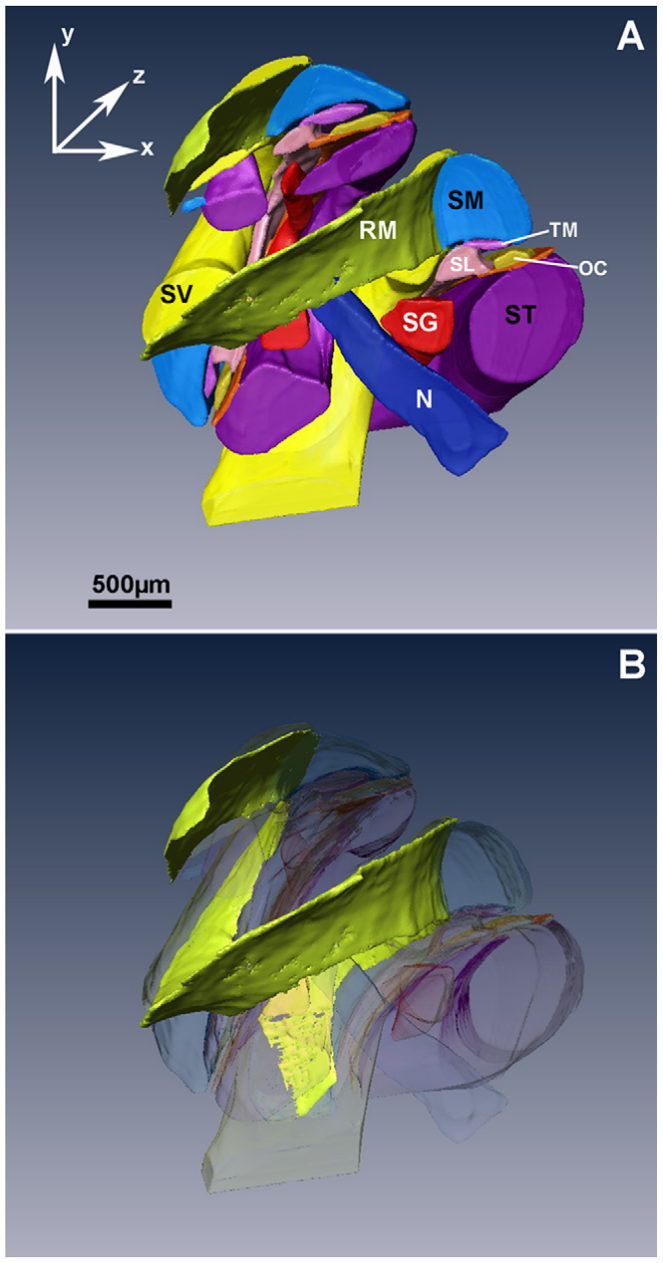
Three dimensional reconstruction of the cochlea. A: Three-dimensional rendered image of the mouse cochlea. The image contains the scala vestibuli (SV), scala media (SM) and scala tympani (ST), Reissner's membrane (RM), tectorial membrane (TM), organ of Corti (OC), spiral limbus (SL), spiral ganglion (SG) and cochlear nerve (N). B: All structures but the Reissner's membrane were made transparent.

Figure 2 shows a corresponding orthogonal plane intercepting at a selected (x,y,z) point. The colored lines in Figure 2 represent the segmentation of the cochlear structures. The segmentation of the structures was used for the 3D-rendering. A rendered 3D-model of the mouse cochlea is presented in Figure 3. Scala vestibuli, scala media and scala tympani are shown in semi-transparent forms, while Reissner's membrane, tectorial membrane, basilar membrane, organ of Corti, spiral lamina, cochlear nerve and the spiral ganglion are presented in opaque colors.

### Length and Cross sectional area measurements

Quantitative data were obtained from the rendered structures. The length of the cochlea according to a line drawn along the pillar heads was 6.67 mm, which is in agreement with published data [e.g. 26].

**Figure 4:**
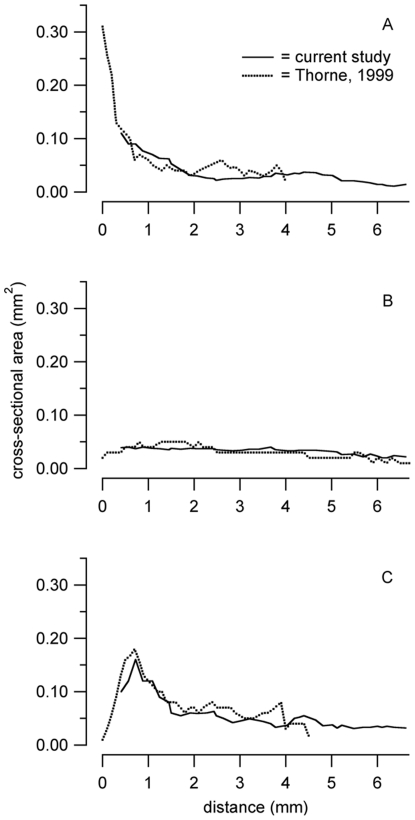
Measurements obtained from the reconstruction. Cross-sectional area as a function of distance from the base of the mouse cochlea are shown by the solid line for A: scala vestibuli, B: scala media, and C: scala tympani. Cross sectional areas decreased from base to apex for scala vestibuli and for scala tympani. The cross sectional area of scala tympani revealed a distinct maximum at about 0.16 mm from the base. Values for scala media cross sectional area vary little. The changes in cross sectional area of scala media are small. For all scalae the cross sectional area measurements, which were obtained in the present study, are in agreement with previously published data [8] and are shown by the broken lines.

The cross-sectional areas of the scala tympani was the largest close to the cochlear base, 0.16 mm^2^, and decreased in the second turn and the apex to 0.031 mm^2^ at its lowest (Fig. 4A). The area of scala media stayed approximately constant at an average of 0.035 mm^2^ along the cochlea (Fig. 4B). For scala vestibuli, the area decreased from 0.11 mm^2^ at the base to 0.011 mm^2^ at its lowest at the apex (Fig. 4C).

## Discussion

Phase contrast imaging with coherent hard X-rays allowed reconstructing soft tissues of a mouse cochlea without removing the cochlear walls or labeling the tissue. The cross-sectional areas of scala tympani, scala media, and scala vestibuli were determined from the reconstructed cochlea and the resulting values for the cross sections are similar to published data obtained with classical histology or MRI. Hence, the experiments demonstrated that quantitative tomography can be achieved with this method. Several benefits exist for using coherent hard X-rays and in-line phase contrast for imaging: no physical manipulation of the cochlear tissue is necessary, the preparation of the tissue is simple, no dehydration is needed, the radiation dose can be small because little absorption of the radiation is required for forming the image, and the submicron resolutions can be achieved. Long periods of scanning time are not necessary for high-resolution images. Scanning time for the present experiments was approximately 20 minutes.

For the current experiment the samples have been decalcified and fixed. Both steps provide ease for the first synchrotron experiment but are not intrinsically necessary. The latter modifications preserve the object's dimensions. This is a strong advantage to other sample modifications, necessary for other methods. For example classical histology needs sample dehydration and inflicts shrinkage on the soft tissue in the order of 25%[e.g. 16], [18].

The dimension obtained from the specimen correlate well with published data on mouse morphology. In this study, the cochlear length was determined with a line drawn along the pillar heads and was 6.67 mm. This value is consistent with previous data, 5.81 to 6.8 mm [26]–[31]. The cross-sectional areas presented here are consistent with the data obtained by Magnetic resonance microscopy, also called high resolution MRI, for scala vestibuli, scala media and scala tympani [8].

Other groups have published reconstructions from the human cochlea using hard X-rays at a synchrotron [3], [22], [32]. Vogel (1999) achieved a resolution of 10 µm but he was not able to image cochlear soft tissue structures. Furthermore, the very thin and hollow lamina spiralis ossea could only be imaged weakly. Glueckert et al. (2011) and Lareida et al,. (2009) were able to image soft tissue structures. Their approach was similar to the one described in this study and the tissue was fixed before imaging. Moreover, osmium tetroxide, a heavy metal was introduced to enhance the contrast for myelinated structures.

Other methods have been implemented that are able to image intact specimen. MRI has been used to determine fluid space volumes, lengths, and cross-sectional areas of the different compartments in the intact mouse cochlea [8]. With this method, Reissner's membrane could not be visualized without the use of a Heidenhain-Susa stain. It has been shown that staining causes substantial shrinkage of the membrane and alters its location within the cochlear spaces [17]. With the present technique, the thin Reissner's membrane can be visualized without the use of stains. Moreover, the use of MRI at high resolutions requires extended times for data collection, in the range of 12–13 hours.

Thin-sheet optical-imaging is another method to image thick samples without physically slicing them and has already been described in 1903 [33]. The plane of light, which is created with a cylindrical lens, is used to illuminate a thin plane within the tissue. The optical section is then observed orthogonal to the plane of light. This method has been refined by others [34]–[36] and has been used to image intact cochleae [5], [37]–[39]. The most recent development uses lasers a light source and allows for non-destructive optical sectioning of the cochlea, thin-sheet laser imaging microscopy (TSLIM) [5], [39]–[41]. With this method, soft tissue structures can be imaged at a cellular resolution. However, TSLIM requires that the tissue is fixed, optically cleared and incubated with a fluorescent probe [39]–[41].

Optical coherence tomography is an interferometric technique that uses typically near infrared light to image soft tissue structures. This technique has been employed to image cochlear structures and to quantify movement patterns of those structures. When interferometry is used to measure the vibration along the optical axis it has a resolution in the nanometer scale. However, the imaging resolution has been reported to be approximately 10 µm [13], [14]. Furthermore, the use of OCT to image cochlear structures requires the opening of the bony cochlear wall.

### Conclusion

A three dimensional model of the mouse cochlea has successfully been created. The values for the structures, which were obtained from the study, are within variation of existing data. The results demonstrate that quantitative tomography is possible in small samples such as the mouse cochlea. Future studies are designed to improve image acquisition and the reconstruction. For the present experiment the specimen was decalcified to identify the contributions of the collagenous part of the cochlear wall on the image formation. For further simplification of the sample preparation, decalcification and fixation of the cochlea are omitted in future experiments.
